# Correlates of self-reported history of mental health help-seeking: a cross-sectional study among individuals with symptoms of a mental or substance use disorder initiating care for HIV in Cameroon

**DOI:** 10.1186/s12888-021-03306-y

**Published:** 2021-06-05

**Authors:** Lindsey M. Filiatreau, Peter Vanes Ebasone, Anastase Dzudie, Rogers Ajeh, Brian Pence, Milton Wainberg, Denis Nash, Marcel Yotebieng, Kathryn Anastos, Eric Pefura-Yone, Denis Nsame, Angela M. Parcesepe

**Affiliations:** 1 Department of Epidemiology, Gillings School of Global Public Health, University of North Carolina at Chapel Hill, Chapel Hill, NC USA; 2 Clinical Research Education Networking and Consultancy, Yaounde, Cameroon; 3 Department of Psychiatry, Columbia University and New York State Psychiatric Institute, New York, NY USA; 4 Institute of Implementation Science in Population Health, Graduate School of Public Health and Health Policy, City University of New York, New York, NY USA; 5 Department of Medicine, Albert Einstein College of Medicine, Bronx, NY USA; 6 Departments of Medicine and Epidemiology & Population Health, Albert Einstein College of Medicine, Bronx, NY USA; 7 Jamot Hospital, Yaounde, Cameroon; 8 Bamenda Regional Hospital, Bamenda, Cameroon; 9 Department of Maternal and Child Health, Gillings School of Global Public Health, University of North Carolina at Chapel Hill, Chapel Hill, NC USA; 10 Carolina Population Center, University of North Carolina at Chapel Hill, Chapel Hill, NC USA

**Keywords:** Mental health, Alcohol use, HIV, Sub-Saharan Africa, Help-seeking, Cameroon

## Abstract

**Background:**

Mental health and substance use disorders (MSDs) increase the risk of poor human immunodeficiency virus (HIV) care outcomes among people living with HIV (PLWH). Receipt of mental health care may improve these adverse outcomes. We aimed to identify correlates of prior mental health help-seeking among PLWH with symptoms of an MSD in Cameroon.

**Methods:**

We characterize prior mental health help-seeking from formal (mental health specialist/general medical provider) and informal (traditional healer/religious leader) sources among 161 people with symptoms of depression (Patient Health Questionnaire-9 scores> 9), anxiety (General Anxiety Disorder-7 scores> 9), probable post-traumatic stress disorder (PTSD Checklist for DSM-5 scores> 30), or possible alcohol use disorder (Alcohol Use Disorders Identification Test scores≥16) who were newly entering HIV care at three healthcare facilities in Cameroon between June 2019 and March 2020. Help-seeking was defined as ever speaking to a formal or informal source about emotional problems, sadness, or the way they were feeling or behaving. We estimated the association between sociodemographic and psychosocial measures and lifetime mental health help-seeking from each type of source using log-binomial regression.

**Results:**

Overall, 55.3% of 161 PLWH with MSD symptoms reported prior mental health help-seeking, with 24.2% and 46.0% seeking help from formal and informal sources, respectively. Religious leaders were the most common source of help (40.4%), followed by general medical professionals (22.4%), traditional healers (16.8%), and mental health specialists (7.4%). Individuals with higher depressive, anxiety, and trauma symptom severity scores were more likely to have sought help than those with lower scores. Individuals with possible alcohol use disorder were the least likely to have sought help. Prior help-seeking was more common among those reporting a higher number of lifetime traumatic events (prevalence ratio [PR]: 1.06; 95% confidence interval [CI]: 1.01, 1.11) and those with a history of emotional intimate partner violence (PR: 1.34; 95% CI: 1.01, 1.80).

**Conclusions:**

Prior mental health help-seeking was associated with psychosocial stressors. Help-seeking from informal networks was more common than formal help-seeking. Training in the provision of evidence-based mental health support for informal networks could improve access to mental health care for PLWH with MSDs in Cameroon.

## Background

Mental health and substance use disorders (MSDs) constitute the largest cause of disability worldwide [1, 2] and increase the risk of early mortality [3–6]. People living with human immunodeficiency virus (HIV) are particularly vulnerable to MSDs, which are among the most common comorbidities among people living with HIV (PLWH) globally [7–9]. MSDs contribute to poor HIV care outcomes (e.g., delayed linkage to care and anti-retroviral therapy [ART] initiation, poor retention in care, and sub-optimal ART adherence) throughout the HIV care continuum [10–12], further increasing risk of early mortality among PLWH with MSDs [13, 14].

Receipt of mental health care not only improves MSD outcomes [15, 16] but has been shown to improve chronic health outcomes in individuals with comorbidities such as HIV in high-income countries [17–19]. In sub-Saharan Africa, where a disproportionate burden of the HIV epidemic is concentrated [20], access to evidence-based mental health care remains limited [21]. To narrow the mental health treatment gap, mental health care task-sharing, or shifting service delivery tasks from health professionals with specialized training to individuals with less specialized, but task-specific training is being scaled-up in numerous settings [22, 23]. This approach has improved access to care and, in many cases, MSD outcomes overall [16, 23–25]. Despite this overall increase in access to mental health care, a substantial proportion of individuals with MSDs still do not have access to evidence-based mental health care in resource-constrained settings [21].

Research suggests that individuals with symptoms of an MSD often seek help from informal support networks such as friends, family members, traditional healers, or religious leaders, instead of, or in addition to, formal care from trained medical providers or mental health specialists [26–28]. In settings where access to formal mental health care remains limited, help-seeking from informal sources is particularly common and could provide a bridge to more formalized, evidence-based care where necessary [29, 30]. While barriers and facilitators of formal and informal mental health help and care-seeking have been identified in high-resource settings [26, 27, 31–34], research from resource-constrained settings is scarce, particularly in populations substantially affected by MSDs such as PLWH [35]. Greater understanding of prior mental health help-seeking behaviors among PLWH with MSD symptoms can clarify which individuals with MSD symptoms have or have not sought help, where they typically turned for help, and who sought informal help but may have benefited from more formal care. Further, this work can identify members of informal support networks who, with additional evidence-based psychoeducation, could serve as key community partners in improving access to and uptake of mental health care broadly. In some settings, these individuals may even aid in screening and referring individuals with symptoms of an MSD to formal care networks.

Given the negative effects of MSDs on quality of life, HIV treatment outcomes, and life expectancy in PLWH in sub-Saharan Africa specifically [10], it is important to understand the mental health help-seeking behaviors of PLWH with symptoms of an MSD [36]. In this study, we characterize prior mental health help-seeking from formal and informal sources in individuals with current symptoms of depression, anxiety, post-traumatic stress disorder (PTSD), or possible alcohol use disorder (AUD). Further, we explore socio-demographic and psychosocial correlates of lifetime mental health help-seeking from formal and informal sources separately, and overall, among individuals with symptoms of depression, anxiety, PTSD, or possible AUD.

## Methods

### Setting

This study was conducted in three public sector health facilities located in the North-West, South-West, and Central (Yaounde) regions of Cameroon. These areas have an estimated HIV prevalence of 5.1%, 3.6%, and 4.4%, respectively [37]. Each site also serves as an International epidemiology Databases to Evaluate AIDS (IeDEA) study site.

### Study design and population

We conducted this structured quantitative interview in a cohort of adults entering care for HIV between June 2019 and March 2020. To be eligible, individuals had to be at least 21 years of age, initiating HIV care at one of the three study sites, and provide written informed consent for participation. Eligible individuals were invited to participate in this study at their first HIV care visit. Individuals transferring HIV care from another clinic were ineligible.

Eligible and consenting individuals were interviewed by a research assistant fluent in French and English. Interviews, which captured data on participants’ socio-demographic and economic backgrounds, mental health, social support, experiences with intimate partner violence (IPV), history of trauma, and substance use were conducted in the participant’s language of choice in a private setting within the health facility. Ethical approval for this study was obtained from the University of North Carolina’s Institutional Review Board and from the national Ethical Committee of Research for Human Health at Yaoundé, Cameroon. This analysis includes data from individuals who screened positive for symptoms of at least one MSD assessed.

## Measures

### Depressive symptoms

The Patient Health Questionnaire-9 (PHQ-9) [38], which has been validated and used among PLWH in sub-Saharan Africa, including in Cameroon [39–42], was used to assess depressive symptoms. Scores range from 0 to 27 with scores greater than 9 considered indicative of moderate or severe depressive symptoms [43]. Cronbach’s alpha from the current study was 0.81.

### Anxiety symptoms

Anxiety symptoms were assessed using the 7-item General Anxiety Disorder-7 (GAD-7) scale [44], which has been validated in a high HIV prevalence population in sub-Saharan Africa [42], and used among PLWH in Cameroon [45]. Scores range from 0 to 21 with scores greater than 9 considered indicative of moderate to severe anxiety symptoms [42, 44]. Cronbach’s alpha from the current study was 0.82.

### Post-traumatic stress disorder

PTSD symptoms were assessed using the PTSD Checklist for DSM-5 (PCL-5) [46, 47] which has been validated and used in high HIV prevalence populations in sub-Saharan Africa [48, 49]. Scores range from 0 to 80 with scores greater than 30 considered indicative of probable PTSD [46]. Cronbach’s alpha from the current study was 0.92.

### Alcohol use disorder

Alcohol use was measured using the Alcohol Use Disorders Identification Test (AUDIT) [50–52], which has been validated and used in high HIV prevalence populations in sub-Saharan Africa [53–55]. Scores range from 0 to 40 with scores equal to or greater than 16 considered indicative of possible AUD [52]. Cronbach’s alpha from the current study was 0.85.

### Comorbid MSD outcomes

We created a dichotomous variable, “MSD comorbidities”, to represent individuals with and without symptoms of two or more of the MSDs assessed (i.e., depression, anxiety, PTSD, AUD).

### Mental health help-seeking

Help-seeking is inconsistently measured throughout the scientific literature, yielding no standard definitions of, or tools for measuring, the outcome of interest [56]. In this study, history of mental health help-seeking was ascertained by asking study participants if they had ever spoken with any of the following individuals about emotional problems, sadness, or the way they were feeling or behaving: 1) traditional healer, 2) religious leader, 3) general medical professional (e.g., general practitioner, nurse), or 4) a mental health specialist (e.g., psychiatrist, psychologist, or social worker). Separate dichotomous variables were created to represent help-seeking from each source (ever/never). Summary variables were created to represent ever help-seeking from 1) any “formal” source (i.e., mental health specialist or general medical provider), 2) any “informal” source (i.e., traditional healer or religious leader), and 3) any formal or informal source.

### Sociodemographic factors

Sociodemographic factors of interest were captured through self-report and included age (continuous), sex, relationship status (single/partnered), education (none/primary/secondary or above), and religion (Protestant/Christian/Born Again/other).

### Potential psychosocial stressors

Potential psychosocial stressors (i.e., trauma and IPV) were also hypothesized as possible correlates of ever seeking mental health help. We assessed exposure to twelve specific potentially traumatic events (PTEs), including lifetime experiences of physical and sexual violence, natural disaster, and loss of a child, among others. One additional question asked individuals to identify any other traumatizing event experienced during childhood or adulthood. These questions were drawn from existing traumatic event screening tools that have been widely used [57–60] and were selected based on their relevance in the given context. The Demographic and Health Survey violence module was used to assess four domains of IPV: controlling behavior, and emotional, sexual, and physical IPV (dichotomous; any/no violence for each domain) [61–63]. We also considered “any IPV” (any controlling behavior, or emotional, sexual, or physical IPV) versus no IPV.

### Statistical analyses

Descriptive statistics (counts/proportions or medians/interquartile ranges [IQRs]) were used to characterize the study population overall and by ever help-seeking. Pearson’s chi-squared tests and Wilcoxon-rank sum tests were used to identify potential correlates of ever help-seeking overall, and from formal and informal sources specifically. Unadjusted log-binomial regression was used to estimate the association between each correlate of interest and ever help-seeking outcome. Multivariable regression was not utilized as the aim of this analysis was to characterize marginal associations as opposed to causal relationships.

## Results

A total of 426 individuals were enrolled in the parent study, with 161 participants (37.8%) reporting symptoms of depression, anxiety, PTSD, or possible AUD and included in this analysis. Of the 161 individuals included, median age was 36 (IQR: 30–43) (Table 1). A majority of participants were female (*n* = 92; 57.1%), had completed some primary school (*n* = 87; 54.0%), and were in a relationship (*n* = 90; 55.9%). Overall, 87 participants (54.0%) reported moderate or severe depressive symptoms, 52 (32.3%) reported moderate or severe anxiety symptoms, 67 (41.6%) screened positive for probable PTSD, and 57 (35.4%) screened positive for possible AUD. Over 40% of included individuals (*n* = 68) screened positive for comorbid MSDs.

**Table 1:** Sociodemographic and economic characteristics of people initiating HIV care with mental and substance use disorder symptoms in Cameroon, overall and by history of mental health help-seeking

	Total ***n*** = 161 N (%)	No mental health help-seeking ***n*** = 72 N (%)	Any mental health help-seeking ***n*** = 89 N (%)	***p***-value
Sex				
Male	69 (42.9)	32 (44.4)	37 (41.6)	0.71
Female	92 (57.1)	40 (55.6)	52 (58.4)	
Age (median/IQR)	36 (30–43)	36 (30–42.5)	37 (31–43)	0.38
Education				
None	16 (9.9)	7 (9.7)	9 (10.1)	0.12
Primary	87 (54.0)	33 (45.8)	54 (60.7)	
Secondary	58 (36.0)	32 (44.4)	26 (29.2)	
Religion				
Catholic	51 (31.7)	26 (36.1)	25 (28.1)	0.30
Protestant	55 (34.2)	26 (36.1)	29 (32.6)	
Born again	43 (26.7)	14 (19.4)	29 (32.6)	
Other	12 (7.4)	6 (8.3)	6 (6.7)	
Relationship status				
Single	71 (44.1)	27 (37.5)	44 (49.4)	0.13
In a relationship/married	90 (55.9)	45 (62.5)	45 (50.6)	

IQR: Interquartile range

Among the included participants, 89 (55.3%) reported ever seeking help from a formal or informal source. Nearly 25% of participants (*n* = 39; 24.2%) reported ever seeking help from a formal source specifically and 50% (*n* = 74; 46.0%) from an informal source specifically. Twenty-four individuals (14.9%) reported ever seeking help from both a formal and informal source. More specifically, 27 (16.8%) individuals reported ever seeking help from a traditional healer, 65 (40.4%) from a religious leader, 36 (22.4%) from a general medical provider, and 12 (7.4%) from a mental health specialist.

**Fig. 1:**
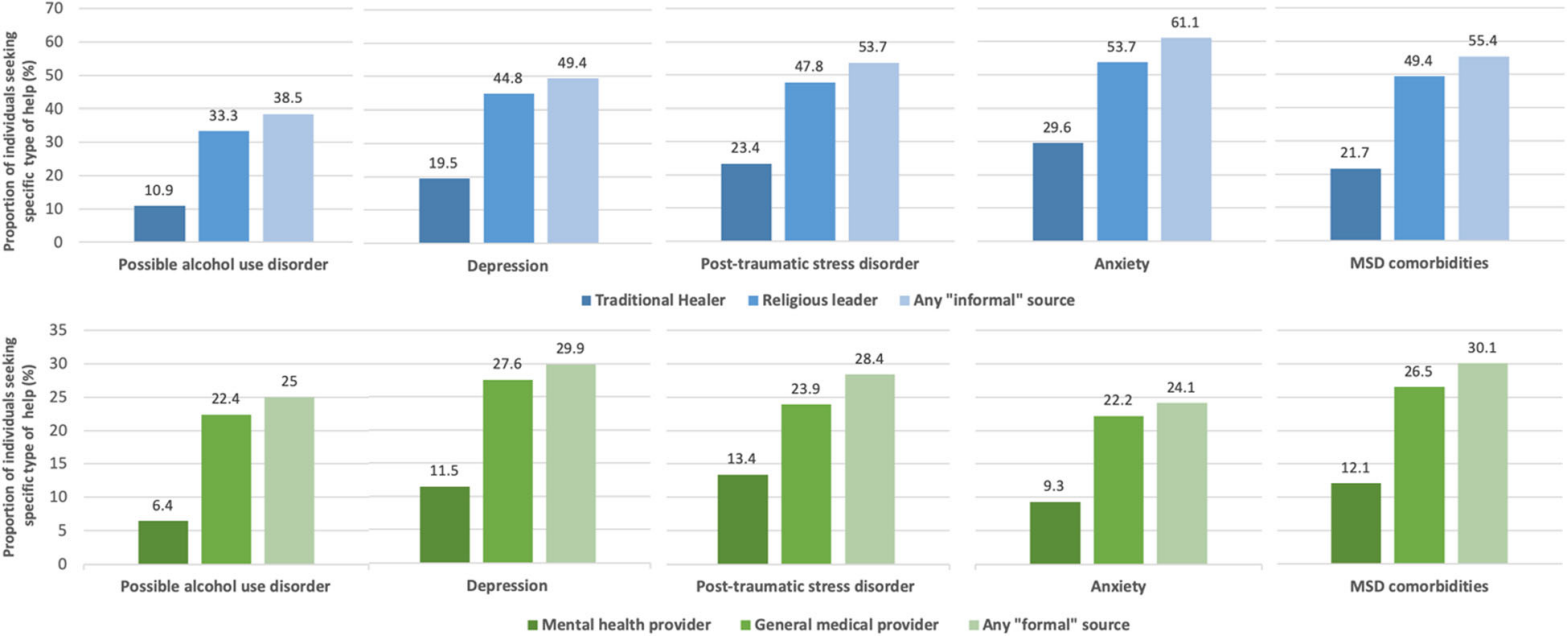
Prevalence of lifetime help-seeking behaviors by symptoms of specific mental health and substance use disorders

The proportions of individuals who reported ever seeking help from a formal or informal source were as follows: 59.8% among those who screened positive for moderate to severe depressive symptoms, 66.7% among those with moderate to severe anxiety symptoms, 61.2% among those with probable PTSD, 49.1% among those with possible AUD, and 64.7% among those with comorbid MSD symptoms. The prevalence of ever seeking help from a mental health specialist, general medical provider, traditional healer, and religious leader specifically, among individuals screening positive for symptoms of each MSD, is presented in Fig. 1.

The prevalence of ever seeking help from a formal or informal source was more common among individuals with higher symptom severity scores on the GAD-7, PCL-5, and PHQ-9 (Tables 2 and 4). The prevalence of ever seeking help from an informal source was more common among individuals with higher symptom severity scores on the GAD-7 and PCL-5 (Table 4). The prevalence of ever seeking mental health help from a formal source was higher among individuals with higher symptom severity scores on the PHQ-9 (Table 4). There was no difference in the prevalence of any type of help-seeking by AUDIT score (Tables 2 and 4).

**Table 2:** Mental and substance use disorder (MSD) symptom scores of people initiating HIV care with MSD symptoms in Cameroon, overall and by history of mental health help-seeking

	Total n = 161 N (%)/median (IQR)	No mental health help-seeking n = 72 N (%)/median (IQR)	Any mental health help-seeking n = 89 N (%)/median (IQR)	p-value
Depressive symptom score	10 (4–13)	9 (3–12.5)	10 (5–14)	0.07
Anxiety symptom score	7 (3–11)	5.5 (2–9.5)	9 (5–11)	< 0.01
PTSD symptom score	26 (11–41)	21.5 (5–37)	27 (14–42)	0.04
Alcohol use score	9 (3–18)	10 (3–19)	9 (2–17)	0.36
MSD comorbidities*				
No	77 (48.4)	44 (61.1)	33 (37.9)	< 0.01
Yes	82 (51.6)	28 (38.9)	54 (62.1)	

MSD: mental and substance use disorders; IQR: Interquartile range; PTSD: post-traumatic stress disorder

*Missing MSD comorbidities *n=2*

Ever seeking help from a formal or informal source was also associated with reporting a history of emotional IPV and reporting a higher number of PTEs (Tables 3 and 4). Ever seeking help from a formal source specifically, and from an informal source specifically, was only associated with reporting a higher number of PTEs (Table 4). Prevalence of ever seeking help from a formal or informal source among those with a history of emotional IPV was 1.34 (95% confidence interval [CI]: 1.01, 1.80) times the prevalence in those with no history of emotional IPV (Table 4). For each additional PTE reported, the prevalence of ever seeking help from a formal or informal source, informal source specifically, and formal source specifically was 1.06 (95% CI: 1.01, 1.11), 1.07 (95% CI: 1.01, 1.14), and 1.15 (95% CI: 1.04, 1.28) times higher, respectively (Table 4).

**Table 3:** Psychosocial stressors among people initiating HIV care with mental and substance use disorder symptoms in Cameroon, overall and by history of mental health help-seeking

	Total n = 161 N (%)/median (IQR)	No mental health help-seeking n = 72 N (%)/median (IQR)	Any mental health help-seeking n = 89 N (%)/median (IQR)	p-value
Emotional IPV				
No	84 (52.2)	44 (61.1)	40 (44.9)	0.04
Yes	77 (47.8)	28 (38.9)	49 (55.1)	
Physical IPV				
No	110 (68.3)	49 (68.1)	61 (68.5)	0.95
Yes	51 (31.7)	23 (31.9)	28 (31.5)	
Sexual IPV				
No	116 (72.0)	55 (76.4)	61 (68.5)	0.27
Yes	45 (28.0)	17 (23.6)	28 (31.5)	
Controlling behavior				
No	32 (20.1)	15 (20.8)	17 (19.5)	0.84
Yes	127 (79.9)	57 (79.2)	70 (80.5)	
Any IPV				
No	23 (14.4)	12 (16.7)	11 (12.5)	0.45
Yes	137 (85.6)	60 (83.3)	77 (87.5)	
Lifetime traumatic events	5 (3–6)	3 (2–6)	5 (3–6)	0.02

*Missing: controlling behavior *n* = 2; Any IPV n = 1

IQR: Interquartile range; IPV :intimate partner violence

**Table 4:** Bivariate associations between correlates of mental health help-seeking among people initiating HIV care with symptoms of a mental or substance use disorder (MSD) in Cameroon

	Any mental health help-seeking PR (95% CI)	Formal mental health help-seeking PR (95% CI)	Informal mental health help-seeking PR (95% CI)
**Mental and substance use disorders**			
Depression score (continuous)	1.02 (1.00, 1.04)	1.06 (1.01, 1.10)	1.02 (0.99, 1.05)
Anxiety score (continuous)	1.04 (1.01, 1.06)	1.04 (0.98, 1.10)	1.05 (1.02, 1.08)
PTSD score (continuous)	1.01 (1.00, 1.02)	1.01 (0.99, 1.02)	1.01 (1.00, 1.02)
Alcohol use score (continuous)	0.99 (0.98, 1.01)	0.99 (0.97, 1.03)	0.99 (0.97, 1.01)
MSD comorbidities (none = ref)	1.34 (1.02, 1.76)	1.30 (0.75, 2.24)	1.44 (1.04, 2.01)
**Psychosocial stressors**			
Any emotional IPV (none = ref)	1.34 (1.01, 1.80)	1.42 (0.81, 2.45)	1.36 (0.97, 1.90)
Any physical IPV (none = ref)	0.99 (0.73, 1.34)	1.08 (0.61, 1.92)	0.91 (0.63, 1.32)
Any sexual IPV (none = ref)	1.18 (0.89, 1.57)	1.01 (0.55, 1.86)	1.24 (0.88, 1.75)
Any controlling behavior (none = ref)	1.04 (0.72, 1.49)	2.14 (0.82, 5.60)	0.88 (0.59, 1.31)
Any IPV (none = ref)	1.18 (0.75, 1.85)	1.96 (0.66, 5.84)	1.06 (0.64, 1.74)
Lifetime traumatic events (continuous)	1.06 (1.01, 1.11)	1.15 (1.04, 1.28)	1.07 (1.01, 1.14)
**Sociodemographic characteristics**			
Sex			
Female	Ref	Ref	Ref
Male	0.95 (0.71, 1.26)	1.03 (0.59, 1.79)	0.81 (0.57, 1.15)
Age (continuous)	1.01 (0.99, 1.02)	1.01 (0.98, 1.04)	1.01 (1.00, 1.03)
Education			
None	Ref	Ref	Ref
Primary	1.10 (0.70, 1.75)	1.78 (0.61, 5.14)	1.01 (0.59, 1.71)
Secondary+	0.80 (0.47, 1.34)	0.64 (0.19, 2.21)	0.76 (0.42, 1.37)
Religion			
Catholic	0.93 (0.64, 1.35)	1.08 (0.57, 2.04)	1.03 (0.64, 1.66)
Protestant	Ref	Ref	Ref
Born again	1.28 (0.92, 1.77)	0.82 (0.39, 1.72)	1.71 (1.14, 2.54)
Other	0.94 (0.51, 1.76)	0.65 (0.17, 2.51)	1.09 (0.52, 2.31)
Relationship status			
Single	1.24 (0.94, 1.63)	1.64 (0.95, 2.84)	1.14 (0.81, 1.59)
Partnered	Ref	Ref	Ref

PR:  prevalence ratio; CI: Confidence interval; PTSD: post-traumatic stress disorder; MSD:  mental and substance use disorders; IPV: intimate partner violence

The prevalence of ever seeking help from a formal or informal source among those with comorbid MSD symptoms was 1.34 (95% CI: 1.02, 1.76) times the prevalence among those without comorbid MSD symptoms (Table 4). The prevalence of ever seeking help from an informal source among those with comorbid MSD symptoms was 1.44 (95% CI: 1.04, 2.01) times the prevalence in those without comorbid MSD symptoms (Table 4). No significant difference was observed in the prevalence of ever seeking help from a formal source among those with and without comorbid MSD symptoms (Table 4).

## Discussion

This study is among the first to characterize self-reported history of mental health help-seeking from formal and informal sources in a population of PLWH with an MSD entering HIV care in a low-resource setting. To our knowledge, it is the first study of this type in Cameroon.

Overall, 55.3% of individuals with an MSD reported ever talking to a formal or informal source about their emotional problems, sadness, or the way they were feeling or behaving. Ever seeking help from an informal source was nearly twice as common as ever seeking help from a formal source, at 46.0% and 24.2%, respectively. Religious leaders were the most common source of help (40.4%), followed by general medical professionals (22.4%), traditional healers (16.8%), and mental health specialists (7.4%). This is likely due in part to the severely limited number of specialized mental health providers across Cameroon, and the low- or no-cost nature of seeking informal help as compared to formal help-seeking. These results are similar to findings from other resource-constrained settings, which suggest help-seeking from informal sources is substantially more common than help- or care-seeking from formal sources [29, 30, 64, 65], and that help-seeking from mental health specialists may be particularly uncommon [64, 66–68]. In a hospital-based study conducted among 384 individuals with mental illness in Ethiopia, more than half of the participants (*n* = 193; 50.2%) had ever sought care from an herbalist or religious healer before seeking formal care services from a psychiatric hospital [30]. Contrary to our findings, help-seeking from general healthcare providers (12.7%) was slightly more common than help-seeking from religious leaders (11.7%) after the onset of post-partum depression among 385 women in Ethiopia [66]. The prevalence of help-seeking from a mental health professional in this population was similar to that found in our study, at 4.2% [66].

Given the high prevalence of help-seeking from informal networks, religious leaders and traditional healers could serve as important partners in improving the overall mental health of PLWH in Cameroon. While some mission hospitals throughout Cameroon already have religious leaders who serve as counselors within the facility, this is not widely implemented. With appropriate psychoeducation and stigma reduction training, members of informal networks could broadly serve as advocates for formal mental health care-seeking, normalizing and promoting this type of care-seeking within their communities, where such care is available. If adequately trained in task-specific care, these individuals could also directly participate in the provision of mental health support, screening individuals for MSDs, and making referrals to formal care as necessary. It is notable that in a cross-sectional study of primary healthcare providers in Cameroon, just 1.8% of surveyed providers knew of a standardized depression screening tool, less than half (49.1%) had received formal mental health training, and just 26.6% reported feeling comfortable working with depressed patients [69]. This suggests that comprehensive evidence-based mental health support and care training is critical not only for informal support networks in Cameroon but could similarly benefit formal care providers.

Higher symptom severity scores on the PHQ-9, GAD-7, and PCL-5, and having symptoms of MSD comorbidities, were significantly associated with ever seeking mental health help from a formal or informal source. This is congruent with help- or treatment-seeking patterns observed in similarly resource-constrained settings [29, 66, 70], and suggests that participants with more severe symptoms, and those with psychiatric comorbidities, are more likely to have sought out mental health help than those with less severe symptoms or symptoms of just one MSD. More severe depressive symptoms were associated with ever seeking help from a formal provider specifically, while more severe anxiety and PTSD symptoms were associated with ever seeking help from an informal provider specifically. This suggests depressive symptoms may be more readily identified by those affected as a medical concern needing attention from a formal provider, while symptoms of PTSD and anxiety may be conceptualized by those affected as less severe or less appropriate to discuss with medical providers in this context. Ensuring informal networks have the skills and training necessary to identify mental illness, particularly severe anxiety and PTSD, as well as psychiatric comorbidities, will be important to bridging the most vulnerable individuals to formal mental health care.

Notably, ever seeking help from a formal or informal source was less commonly reported among individuals screening positive for possible AUD when compared to individuals with current symptoms of depression, anxiety, or PTSD. No significant association was observed between AUDIT scores and any of the help-seeking behaviors explored, suggesting AUD may function differently than mood or anxiety disorders within this context. For example, unhealthy drinking may be viewed as a more normalized behavior within society, less concerning by those affected, or less appropriate for discussion with formal or informal networks among PLWH with possible AUD in Cameroon. Alternatively, it is possible that unhealthy drinking is more stigmatized than mood or anxiety disorder symptoms, creating greater hesitancy to disclose concerns around alcohol use to formal or informal networks. Help-seeking for AUD may be further hindered by limited availability or perceived ineffectiveness of substance use-related services. This is consistent with findings from studies in similar contexts [68, 71]. For example, just 6 of 57 (10.5%) individuals screening positive for harmful alcohol use in a study of 1500 adults in Ethiopia had sought help from a health center for their alcohol use [71]. The primary barriers to help-seeking were wanting to handle the problem on their own (63.3%), believing the problem would get better by itself (60.2%), being unsure of where to go for help (57.0%), and being unbothered by the problem (49.2%) [71]. Additional research into alcohol use and treatment norms could shed light on the drivers of low help-seeking among PLWH with symptoms of possible AUD in this context.

Current socio-demographic factors were not significantly associated with any type of ever help-seeking, with the exception of increased help-seeking from informal sources in individuals who identified as Born Again. We hypothesized socio-demographic factors would be associated with help-seeking due to their relationship with poor mental health and HIV care outcomes [7], and existing literature suggesting that age, sex, education, or income may influence help-seeking [35, 72–75]. However, our results are consistent with studies from similarly low-resourced settings [67, 70]. For example, in a study conducted in rural India, no association was observed between gender, religion, education, age, caste, or employment status and treatment-seeking [70].

Psychosocial stressors, specifically higher numbers of reported PTEs and history of emotional IPV were associated with mental health help-seeking. In a study from Nigeria, emotional IPV was also associated with an increased likelihood of help-seeking while sexual IPV was not [65]. In this setting, physical IPV was associated with increased help-seeking as well, contrary to our findings [65]. It is possible that emotional IPV functions differently from physical and sexual IPV in Cameroon due to cultural or gender norms within relationships. For example, if physical and sexual violence are normalized behaviors within partnerships, or are viewed as problems that should be kept private, individuals may be less inclined to seek help for distress resulting from this type of IPV [76, 77]. Alternatively, individuals may fear seeking help if they have experienced physical or sexual IPV and are concerned about re-victimization [78], or may find it more appropriate to seek general medical care for physical problems as opposed to emotional distress resulting from physical or sexual violence. Our results suggesting greater trauma is associated with a greater likelihood of help-seeking are consistent with existing literature [75, 79]. Overall, findings highlight the significance of co-occurring psychosocial stressors in understanding patterns of mental health help-seeking.

Results from this study should be interpreted within the context of study limitations. Given the cross-sectional nature of these data, we were unable to investigate the longitudinal effects of socio-demographic and psychosocial factors on mental health help-seeking behaviors. Further, data were not available on the timing, frequency of, or reasoning for prior help-seeking behaviors. Importantly, this study captured information on lifetime help-seeking and current MSD symptoms. Thus, we are unable to assess to what extent participants’ MSD symptoms at the time of help-seeking were similar or dissimilar to their current MSD symptoms. However, evidence suggests symptoms of depression, anxiety, PTSD, and AUD are often chronic or reoccurring throughout one’s lifetime [80, 81]. Because we defined help-seeking as ever speaking to a source about their emotional problems, we cannot draw conclusions about efforts to seek help that may have been unsuccessful. This is particularly relevant to formal help-seeking as it is possible participants tried to seek formal help but were ultimately unable to speak with someone due to highly limited provider availability. Further, these results are subject to social desirability bias and recall bias as reported in other studies on the agreement between self-report and medical record care-seeking [82, 83]. Finally, participants were recruited from three clinics specifically selected because of existing data collection capacity, limiting the generalizability of findings to other settings or populations. Future studies should investigate MSD symptoms, help-seeking behaviors, and predictors of help-seeking over time in order to ascertain causal relationships between key factors of interest and mental health help-seeking among PLWH as they move through the HIV care continuum.

## Conclusions

Increased access to formal and informal mental health help is needed in Cameroon given just over half of PLWH with symptoms of an MSD in this sample have previously sought help. History of mental health help-seeking from informal networks was more common than formal help-seeking. Seeking help from formal or informal sources, and from formal and informal sources separately, was significantly associated with psychosocial stressors. Psychoeducation and training in the provision of evidence-based mental health support for informal networks have the potential to improve access to mental health care for PLWH with less severe symptoms and improve referral to formal services for those with more severe symptoms and substantial care needs.

## Data Availability

Data used in this analysis are not publicly available at the present time. Data may be made available by author AP on reasonable request.

## Abbreviations

ART: Anti-retroviral therapy
AUD: Alcohol use disorder
AUDIT: Alcohol Use Disorders Identification Test
CI: Confidence interval
GAD-7: General Anxiety Disorder-7
HIV: Human immunodeficiency virus
IeDEA: International epidemiology Databases to Evaluate AIDS
IPV: Intimate partner violence
IQR: Interquartile range
MSD: Mental or substance use disorder
PCL-5: PTSD Checklist for DSM-5
PHQ-9: Patient Health Questionnaire-9
PLWH: People living with HIV
PR: Prevalence ratio
PTE: Potentially traumatic event
PTSD: Post-traumatic stress disorder
